# Neurotoxicity and nephrotoxicity caused by combined use of lithium and risperidone: a case report and literature review

**DOI:** 10.1186/s40360-016-0101-x

**Published:** 2016-12-14

**Authors:** Chih-Wei Hsu, Yu Lee, Chun-Yi Lee, Pao-Yen Lin

**Affiliations:** 1 Department of Psychiatry, Kaohsiung Chang Gung Memorial Hospital and Chang Gung University College of Medicine, Kaohsiung, Taiwan; 2 Department of Psychiatry, Kaohsiung Chang Gung Memorial Hospital, 123, Dapi Road, Niaosong District, Kaohsiung City 833, Taiwan

**Keywords:** Risperidone, Lithium, Neurotoxicity, Acute kidney injury, Drug-drug interaction

## Abstract

**Background:**

Combination lithium, a mood stabilizer, and risperidone, an atypical antipsychotic drug, is widely used for treatment of psychotic disorders. Rare reports concern severe adverse drug reaction in multiple organic systems with their combined use. We report two episodes of neurotoxicity and nephrotoxicity in a patient following the combined use of lithium and risperidone.

**Case presentation:**

A 55-year-old male had a diagnosis of schizoaffective disorder at the age of 51. He was initially treated with a combination of lithium and olanzapine 5 to 15 mg/day for 2 years. He was admitted to psychiatric ward at the age of 53 due to manic episode with psychotic feature. Because of poor blood sugar control, we switched olanzapine 20 mg/day to risperidone 4.5 mg/day with combination of lithium 900mg/day. The patient presented neurotoxicity, neuroleptic-malignant-syndrome like symptoms, and nephrotoxicity, elevation of blood creatinine and decreased urine output few days later. These signs were fully recovered within 2 days after we discontinued all medications and gave normal saline hydration. Then we re-administered decreased dosage of lithium 600 mg/day and risperidone 3 mg/day, and the similar episode occurred again 3 days later. All drugs were discontinued again, then his delirium resolved and abnormal data returned to normal 1 day later. Finally, the patient was treated with risperidone 2 mg/day as monotherapy, and no episode of neurotoxicity and nephrotoxicity appeared in the following 2 years.

**Conclusions:**

The case exemplifies neurotoxicity and nephrotoxicity after combined use of lithium and risperidone. These adverse effects resolved soon after discontinuing these medications and adequate hydration. Clinicians should be cautious about neurological and renal adverse effects.

## Background

Lithium is a mood stabilizer for treatment of bipolar disorders [1]. Risperidone, an atypical antipsychotic drug with both dopamine D_2_ antagonistic and serotonin 5-HT_2A_ antagonistic properties, is widely used for treatment of psychotic disorders. Combination of lithium and risperidone is commonly used for bipolar disorder or schizoaffective disorder [2]. There are rare reports concerning severe adverse drug reaction, neurotoxicity, with their combined use (Table 1) [3–9]. We report a patient who presented not only neurotoxicity but also nephrotoxicity following the combined use of lithium and risperidone.

**Table 1:** Seven previous reports and 2 episodes of neurotoxicity and nephrotoxicity in our patient caused by combined use of lithium and risperidone

R.N.	Age	Sex	Diagnosis	Risperidone dose (mg)	Lithium dose (mg)	Time to onset^a^	Clinical presentation	Laboratory values and Image
[3]	69	F	BD	6	450	3 weeks	Delirium	Cr: NA; Li: 1.36
[4]	30	M	BD	NA	900	1 week	NMS	Cr: 1.6; Li: 0.6; CPK: 1428
[5]	46	M	NA	3	900	2 years	NMS, DKA	BUN: 66
[6]	54	F	BD	3	1500	2 months	NMS	Cr: NA; Li: 0.7; CPK: 791
[7]	60	F	SD	6	NA	3 weeks	Delirium	EEG: Abnormal; CK: 953
[8]	75	M	BD	2	900	4 days	Lethargy	EEG: Abnormal; Cr: NA; Li: 1.47
[9]	60	F	SD	6	NA	6 days	NMS	EEG: Abnormal; CPK: 953
I^b^	56	M	SD	4.5^c^	900	12 days	Delirium	BUN/Cr: 24/1.33; Li: 1.42; CK: 975
II^b^				3	600	3 days	Delirium	BUN/Cr: 29/1.36; Li: 1.54; CK: 90

^a^Time is calculated from beginning the combination of lithium and risperidone to the occurrence of adverse events

^b^I and II are serial numbers of the 2 episodes of our patient during hospitalization

^c^IM Risperidone 25 mg/vial was prescribed 7 days before oral risperidone

## Case presentation

A 55-year-old male had a diagnosis of schizoaffective disorder at the age of 51. He was initially diagnosed with bipolar I disorder at the age of 30, and mainly treated with a combination of lithium and valproic acid from age 30 to 51. At the age of 51, he was observed to have a manic episode concurrent with persecutory delusions and auditory hallucination. These psychotic symptoms persisted for more than 3 weeks after his manic symptoms had subsided. Later, a combination of lithium and olanzapine 5 to 15 mg/day were given as the main treatment. In addition, he had history of type 2 diabetes mellitus and hyperlipidemia for more than 10 years. He did not have any substance use or neurological disorder before. This time, after poor drug adherence for 4 months, he was admitted to our ward at the age of 53, due to symptoms including elevated mood, increased goal-directed activity, talkativeness, decreased need for sleep, and grandiose delusion lasting for 1 week.

Lithium 600 mg/day and olanzapine 10 mg/day were initially prescribed, and the dosages were titrated to 900 mg/day and 20 mg/day, respectively, over 1 week. His lithium serum level was reported to be 0.60 mEq/L (day 4) and 0.58 mEq/L (day 14). Because of poor blood sugar control, intramuscular injection of risperidone 25 mg was prescribed for switching antipsychotic agent on day 15. Olanzapine was discontinued and risperidone 4.5 mg/day was started on days 22 to 24. However, on day 27, the patient presented slurred speech, muscle rigidity, delirium (confused consciousness, inattention, and disorientation), and decreased urine output. At the same time, he did not present fever, focal neurological signs, or gastrointestinal symptoms. Laboratory data showed: blood urea nitrogen (BUN; 24 mg/dL), creatinine (1.33 mg/dL), serum lithium (1.42 mEq/L) on day 28, and creatine kinase (975 U/L) on day 29, compared with BUN (8 mg/dL) and creatinine (0.8 mg/dL) on admission. All medications were discontinued and he was treated with normal saline hydration. The delirium and abnormal laboratory data were fully recovered within 2 days, as follows: BUN (12 mg/dL), creatinine (0.82 mg/dL), and serum lithium (0.82 mEq/L). Lithium 600 mg/day and risperidone 3 mg/day were re-administered on day 32. But symptoms of delirium and elevated BUN (29 mg/dL), creatinine (1.36 mg/dL), serum lithium (1.54 mEq/L), and creatine kinase (90 U/L) were noted on day 35. All drugs were discontinued again and fluid hydration was given. One day later, the delirium resolved and abnormal data returned to normal: BUN (16 mg/dL), creatinine (0.88 mg/dL), and serum lithium (1.06 mEq/L). Finally, risperidone 2 mg/day monotherapy was given on day 38. The patient was discharged in a stable condition on day 42. He was kept regular treatment with risperidone 2 to 3 mg/day, and no episode of neurotoxicity and nephrotoxicity appeared in the following 2 years.

## Conclusions

Our patient had 2 episodes of neurotoxicity and nephrotoxicity during this hospitalization. He presented delirium, acute kidney injury (AKI) with increased level of serum creatinine [10], and lithium intoxication, which had developed soon after combined administration of lithium and risperidone, and subsided after discontinuing these medications. There were no other obvious medical causes that could better account for the development of above symptoms in this patient. The switching of antipsychotics was suitable for our patient with history of type 2 diabetes mellitus and hyperlipidemia [11]. His clinical features were considered to be side effects of lithium-risperidone interaction. The rating on the Naranjo Adverse Drug Reaction Probability Scale was 10, considered a “definite” adverse effect [12].

We searched PubMed from inception to June 2016 for literature review of neurotoxicity and nephrotoxicity due to combined use of lithium and risperidone. Google Scholar and pharmacovigilance databases of UK, Dutch, Australian, and Canadian were also searched for additional relevant articles. The search terms were: “lithium” and “risperidone” and (neuroto* or nms or neuroleptic malignant syndrome or delirium) as Search 1; lithium and risperidone and (nephroto* or renal failure or kidney injury) as Search 2. Articles without the direct concomitant use of lithium and risperidone or with toxicity highly related to other physical problem or obscure support were excluded. The Search 1 and 2 finally yielded 7 articles (twenty results from PubMed, then 14 articles were excluded and 1 report were included from Google Scholar) and showed in Table 1 [3–9]. These cases showed the mean age of 56.3 years, and no gender difference (4 females). The doses of lithium and risperidone ranged from 450 to 1500 mg/day and 2 to 6 mg/day, respectively, which are within normal therapeutic range. Side effects were noted within 3 weeks (5 of 7 cases) after the initial combination of lithium and risperidone, and varied from 4 days to 2 years. All cases developed neurotoxicity with symptoms of lethargy, delirium, and even neuroleptic malignant syndrome (NMS) (4 of 7 cases). NMS is characterized by delirium, muscular rigidity, fever, and autonomic nervous system dysregulation with typically high levels of creatine phosphokinase but not necessary in elevation of creatinine [13, 14]. Furthermore, our report specially showed the change of renal function in detail compared to previous reports.

There are two hypotheses of neurotoxicity from drug-drug interaction between lithium and risperidone. One is pure lithium intoxication-induced neurotoxicity without a relationship with risperidone [8]. The other one is that both lithium and risperidone increase the dopamine receptor blockade. Lithium inhibits pre-synaptic dopamine release, and risperidone blocks dopamine receptors [9]. As for nephrotoxicity, there are inconsistent results. One study including 13 patients indicates that switching a combination of lithium plus a conventional antipsychotic to a combination of lithium plus risperidone within 9 days is generally well tolerated and no statistically significant differences in the pharmacokinetics of serum lithium [15]. The other studies support lithium and risperidone may each induce it. Lithium may impair tubular function and next result in several renal complications, including AKI [16, 17]. Risperidone was also described as increasing the risk of AKI in a population-based cohort study [18]. Our case is an indicator to prove the nephrotoxicity by the concomitant use of these pharmaceuticals. He did not have a history of AKI while being treated with lithium or risperidone alone. We assume his AKI have a “2-pronged” effect of nephrotoxicity, which causes functional impairment of nephrons.

Based on the literature review and our report, close observation of neurological signs for at least 3 weeks is necessary when lithium and risperidone are used at the same time. Once neurotoxicity occurs, further monitoring of renal function for nephrotoxicity survey is also required. Discontinuance of these agents will lead to remission of the aforementioned adverse effects.

## Abbreviation

BD: Bipolar disorder
BUN: Blood urea nitrogen
CPK: Creatine phosphokinase
Cr: Creatinine
DKA: Diabetic ketoacidosis
EEG: Electroencephalography
Li: Lithium
NA: Not available
NMS: Neuroleptic malignant syndrome
R.N.: Reference number
SD: Schizoaffective disorder

## References

[1] Oruch R, Elderbi MA, Khattab HA, Pryme IF, Lund A (2014). Lithium: a review of pharmacology, clinical uses, and toxicity. Eur J Pharmacol.

[2] Mensink GJ, Slooff CJ (2004). Novel antipsychotics in bipolar and schizoaffective mania. Acta Psychiatr Scand.

[3] Chen B, Cardasis W (1996). Delirium induced by lithium and risperidone combination. Am J Psychiatry.

[4] Bourgeois JA, Kahn DR (2003). Neuroleptic malignant syndrome following administration of risperidone and lithium. J Clin Psychopharmacol.

[5] Ananth J, Johnson KM, Levander EM, Harry JL (2004). Diabetic ketoacidosis, neuroleptic malignant syndrome, and myocardial infarction in a patient taking risperidone and lithium carbonate. J Clin Psychiatry.

[6] Boker H, Brandenberger M, Schopper C (2007). [Neurotoxicity related to lithium-risperidon combination treatment in a patient with schizoaffective disorder]. Psychiatrische Praxis.

[7] Kosehasanogullari SG, Akdede B, Akvardar Y, Akan M, Tunca Z (2007). Neuroleptic malignant syndrome caused by combination of risperidone and lithium in a patient with multiple medical comorbidities. Prog Neuro-Psychopharmacol Biol Psychiatry.

[8] Boora K, Xu J, Hyatt J (2008). Encephalopathy with combined lithium-risperidone administration. Acta Psychiatr Scand.

[9] Boeker H, Seidl A, Schopper C (2011). Neurotoxicity related to combined treatment with lithium, antidepressants and atypical antipsychotics. Schweizer Archiv Fur Neurologie Und Psychiatrie.

[10] Khwaja A (2012). KDIGO clinical practice guidelines for acute kidney injury. Nephron Clin Pract.

[11] Grande I, Bernardo M, Bobes J, Saiz-Ruiz J, Alamo C, Vieta E (2014). Antipsychotic switching in bipolar disorders: a systematic review. Int J Neuropsychopharmacol.

[12] Naranjo CA, Busto U, Sellers EM, Sandor P, Ruiz I, Roberts EA, Janecek E, Domecq C, Greenblatt DJ (1981). A method for estimating the probability of adverse drug reactions. Clin Pharmacol Ther.

[13] Tse L, Barr AM, Scarapicchia V, Vila-Rodriguez F (2015). Neuroleptic malignant syndrome: a review from a clinically oriented perspective. Curr Neuropharmacol.

[14] Baeza-Trinidad R, Brea-Hernando A, Morera-Rodriguez S, Brito-Diaz Y, Sanchez-Hernandez S, El Bikri L, Ramalle-Gomara E, Garcia-Alvarez JL (2015). Creatinine as predictor value of mortality and acute kidney injury in rhabdomyolysis. Intern Med J.

[15] Demling J, Huang ML, Remmerie B, Mannaert E, Sperling W (2006). Pharmacokinetics and Safety of Combination Therapy with Lithium and Risperidone. Pharmacopsychiatry.

[16] Oliveira JL, Silva Junior GB, Abreu KL, Rocha Nde A, Franco LF, Araujo SM, Daher Ede F (2010). Lithium nephrotoxicity. Rev Assoc Med Bras.

[17] Rej S, Shulman K, Herrmann N, Harel Z, Fischer HD, Fung K, Gruneir A (2014). Prevalence and correlates of renal disease in older lithium users: a population-based study. Am J Geriatr Psychiatry.

[18] Hwang YJ, Dixon SN, Reiss JP, Wald R, Parikh CR, Gandhi S, Shariff SZ, Pannu N, Nash DM, Rehman F (2014). Atypical antipsychotic drugs and the risk for acute kidney injury and other adverse outcomes in older adults: a population-based cohort study. Ann Intern Med.

